# Oral Supplementation with Modified Natural Clinoptilolite Protects Against Cadmium Toxicity in ICR (CD-1) Mice

**DOI:** 10.3390/toxics13050350

**Published:** 2025-04-27

**Authors:** Michaela Beltcheva, Yana Tzvetanova, Peter Ostoich, Iliana Aleksieva, Tsenka Chassovnikarova, Liliya Tsvetanova, Rusi Rusew

**Affiliations:** 1 Institute of Biodiversity and Ecosystem Research, Bulgarian Academy of Sciences, 1 Tsar Osvoboditel Blvd., 1000 Sofia, Bulgaria; p.ostoich@gmail.com (P.O.);; 2 Institute of Mineralogy and Crystallography Acad. I. Kostov, Bulgarian Academy of Sciences, Acad. G. Bonchev Str., bl. 107, 1113 Sofia, Bulgaria; yana.tzvet@gmail.com (Y.T.); lilicvetanova79@abv.bg (L.T.); r.rusev93@gmail.com (R.R.); 3 Department of Zoology, Faculty of Biology, Plovdiv University, 4 Tzar Assen Str., 4000 Plovdiv, Bulgaria

**Keywords:** clinoptilolite, mice, cadmium toxicity, clinoptilolite detoxification, hematological parameters, oxidative stress, micronuclei frequency

## Abstract

For the first time, this study investigates in vivo the potential of Na-modified natural clinoptilolite to mitigate cadmium toxicity in ICR mice, a model relevant to human health. We enhanced natural clinoptilolite to improve its cadmium (Cd) exchange capacity. Mice were exposed to environmentally realistic cadmium nitrate Cd(NO_3_)_2_ doses in their drinking water. The detoxification efficacy of the mineral was evaluated over 45 days in four groups: control (no supplementation), Cd(NO_3_)_2_ only, clinoptilolite only, and a combination of Cd(NO_3_)_2_ and clinoptilolite. We assessed Cd bioaccumulation in the liver and kidneys, genotoxicity (micronucleus assay), hematological parameters, and oxidative stress markers. Cd exposure resulted in significant bioaccumulation, reduced growth, changes in erythrograms, DNA damage, and oxidative stress. Mice receiving clinoptilolite alone showed a significant increase in body mass. Modified clinoptilolite led to a nearly 48% reduction in Cd accumulation and a 30% increase in Cd excretion in the Cd-plus-clinoptilolite group compared to the Cd-only group. Erythrogram and leukogram parameters returned to near-normal levels, with reductions in malondialdehyde (MDA) and increases in glutathione (GSH) observed by the end of the experiment. No elevated levels of micronuclei were found following clinoptilolite supplementation. These results suggest that modified clinoptilolite may be a cost-effective detoxifier in Cd-polluted regions.

## 1. Introduction

Heavy metals have numerous sources of exposure, routes, and distinct biotransformation and elimination pathways [1]. In recent years, natural zeolites have gained popularity as an effective solution for metal removal due to their advantageous properties and structural characteristics. Consequently, they are utilized across various industrial, agricultural, environmental, and biological applications [2,3].

**Figure 1:**
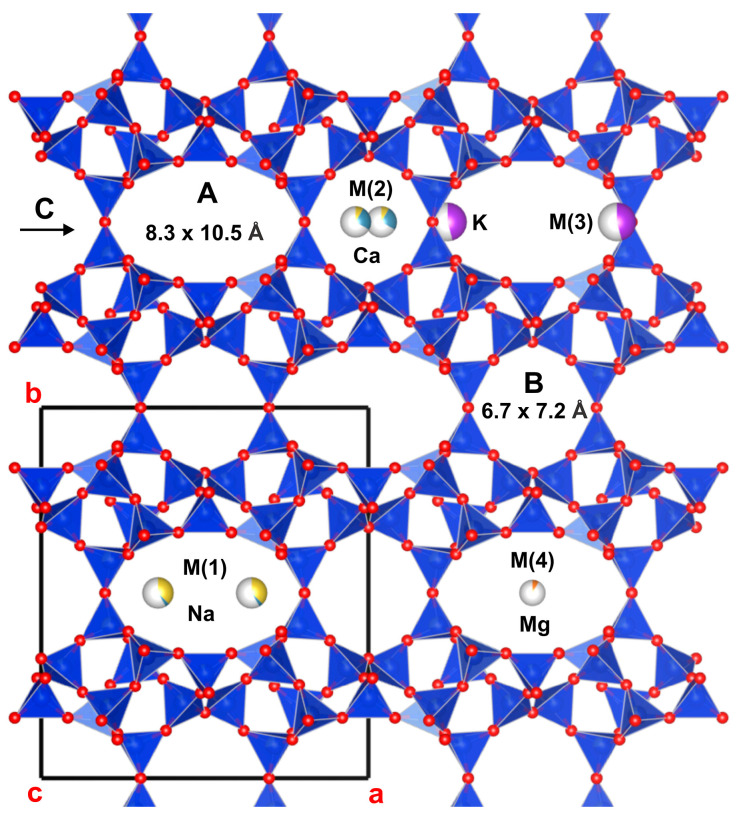
The crystal structure of clinoptilolite, with channels (A, B and C) and cation positions (M1, M2, M3 and M4).

Zeolites are a large group of minerals that are hydrous framework aluminosilicates and contain exchangeable cations, primarily sodium, calcium, and potassium, and less commonly magnesium, barium, and strontium. The most abundant natural zeolite is clinoptilolite, which comprises clinoptilolite–calcium (Ca), clinoptilolite–potassium (K), and clinoptilolite–sodium (Na). The minerals of the clinoptilolite series possess a heulandite (HEU)-type framework topology and silicon (Si)/aluminum (Al) ratio greater than 4 [4]. They have monoclinic symmetry, space group *C*2/*m*, which may be lowered to *C*2 or *Cm* [4,5]. Their HEU-type topology is characterized by a two-dimensional system of three types of channels, which are defined by ten- and eight-membered tetrahedral rings. The large, ten-membered A channel and the smaller, eight-membered B channel are parallel to the *c*-axis, and both intersect the eight-membered C channel, which is parallel to the *a*-axis [6,7,8,9]. The channels are occupied by extra-framework cations [(Na^+^, K^+^, Ca^2+^, magnesium (Mg)^2+^, barium (Ba)^2+^, and strontium (Sr)^2+^], which are coordinated by H_2_O molecules in irregular polyhedral and/or framework oxygens [10]. Four extra-framework cation positions are recognized in the tetrahedral framework of HEU-type zeolites: M (1), M (2), M (3), and M (4) [8] (Figure 1). The wide variability of the cation exchange properties of clinoptilolite depends on the Si/Al ratio, the specific position of the extra-framework cations within the structure, their coordination by H_2_O molecules, and their interactions with framework oxygens [10,11].

In Bulgaria, sizable deposits of high-quality zeolites, mainly clinoptilolite, are present in the eastern parts of the Rhodope Mountains. Five clinoptilolite deposits and one mordenite deposit were identified and explored by drilling. They are related to the Paleogene volcanism’s first and second Rupelian acid volcanic phases [12,13,14,15]. Given the focus of this study on natural clinoptilolite from the Beli Plast deposit, the specific zeolite mineral will henceforth be referred to as “clinoptilolite”.

Natural zeolites possess unique qualities that may render them attractive alternatives to currently available chelating agents for removing heavy metals from blood, tissues, and adipose stores [16]. Natural clinoptilolite is not a chelator in the traditional sense; instead, it operates through ion exchange and adsorption. Due to its low cost, natural clinoptilolite has been the focus of numerous in vitro studies exploring its sorption properties and interactions with biological systems at the cellular and molecular levels. In contrast, in vivo studies examining the heavy metal sorption capacity of natural clinoptilolite in biological models (specifically laboratory mice) are scarce and mainly focused on the removal of lead (Pb) [17,18,19]. The results indicate that 30% to 70% of the ingested Pb is adsorbed by clinoptilolite in the gastrointestinal tract and subsequently excreted from the body in the feces [17,18,19]. Ref. [20] suggests that clinoptilolite does not impact the organism’s homeostasis of trace elements but shows notable selectivity for heavy metals and toxic substances. There are assumptions and indirect evidence that the mineral influences the redox status of the organism based on its well-defined ion exchange potential [20,21,22].

The primary parameters used to assess organisms’ health status and provide an early warning of potentially harmful physiological changes due to xenobiotic exposure include heavy metal bioaccumulation, hematological indices, oxidative stress, and DNA damage assessments. The pattern of Cd bioaccumulation results in toxicity, which can potentially cause adverse effects in organisms, including growth inhibition [23], oxidative and mitochondrial stress, and apoptosis [24,25]. Cd is known to have a long half-life [26]. Following absorption, Cd accumulates primarily in the liver and kidneys, presenting a significant risk of severe hepatotoxicity and nephrotoxicity [27,28]. The liver is a major target organ during acute Cd exposure, while the kidneys are significantly affected by prolonged exposure to Cd [29]. The enterohepatic circulation of Cd induces the synthesis of metallothionein (MT) and the formation of Cd-MT complexes, preventing Cd from reacting with target molecules [30]. This makes Cd less accessible for sorption, and the effectiveness of clinoptilolite may depend on its ability to release Cd from these complexes.

A comprehensive investigation into the impact of heavy metals on organisms indicates that blood may serve as an effective biomarker for Cd exposure and toxicity in vivo [1] due to its accessibility and the range of measurable parameters, which makes it a powerful tool. Hematological parameters are frequently used in mammalian medicine. Cd has been demonstrated to cause significant alterations in blood biomarkers in mice [31,32,33], including a reduction in hemoglobin (Hb), hematocrit (Hct), and red blood cell (RBC) count, along with increases in white blood cell (WBC) count [34,35,36]. Only a few studies have reported changes in hematological indices, such as leukocyte and thrombocyte counts, during hemoperfusion with clinoptilolite [18,20].

Oxidative stress caused by generating reactive oxygen species (ROS), including superoxide ions, hydrogen peroxide, and hydroxyl radicals, is considered a significant mechanism underlying Cd-induced toxicity [37]. This process compromises antioxidant defense mechanisms and increases ROS production by mitochondria as by-products of oxidase activities [38]. Furthermore, studies show that Cd impacts glutathione (GSH) levels, a tripeptide that plays a crucial role in non-enzymatic antioxidant protection. Research indicates that Cd initially depletes GSH stores by inhibiting GSH synthesis and enhancing GSH oxidation [37]. This process acts as the first line of defense against Cd-induced toxicity [39]. Clinoptilolite has shown promise in in vitro studies for reducing oxidative stress, primarily through its ability to eliminate heavy metals and its potential to influence antioxidant enzyme activity [21,22].

The formation of micronuclei (MNs) has been extensively utilized as a biomarker of genotoxic stress and genetic instability in many human and non-human models [40]. It has been demonstrated that MN formation is elevated in the peripheral blood erythrocytes of mice residing in areas contaminated with Cd [41,42]. The prolonged exposure to elevated concentrations of Cd exerts genotoxic effects on peripheral blood and bone marrow cells in rats, such as DNA damage, leading to mutations, chromosomal aberrations, and the formation of MNs [43,44,45]. Their mechanisms include inhibiting DNA repair and direct reactivity with DNA phosphate groups [46]. Direct research assessing the influence of clinoptilolite on MN formation is lacking; however, some studies touch upon the genoprotective potential of clinoptilolite, which is relevant to MN formation [47].

The slow elimination rates and biomagnification of Cd in higher levels of food chains in ecosystems [47,48] are expected to create serious problems in the next decade [49]. Consequently, the ongoing challenge of removing accumulated Cd remains a significant concern [50]. The long-term effects of clinoptilolite on biological systems, including its potential toxicity and biocompatibility, must be thoroughly investigated. In this regard, conducting an in vivo study incorporating appropriately activated natural clinoptilolite as a Cd^2+^ sorbent in eco-toxicological experiments with model mammal organisms warrants further exploration. The potential of modified natural clinoptilolite to detoxify Cd in mammalian organisms must be thoroughly evaluated through oral administration due to the harsh conditions of the gastrointestinal tract, including varying pH levels and digestive enzymes. Clinoptilolite’s ability to reduce heavy metal absorption in the gastrointestinal tract is crucial for human health, and studies that focus on oral administration may be more relevant to human applications. Accordingly, this study aimed to enhance the Cd^2+^ exchange capacity of natural clinoptilolite from the “Beli Plast” deposit through Na activation and to evaluate its protective efficacy as a Cd sorbent to reduce hepatic and renal Cd bioaccumulation, Cd-induced oxidative stress, hematological alterations, and genotoxic damage in ICR mice. The formulation of the resulting modified clinoptilolite is of the utmost importance, as it must incorporate elements deemed safe for utilization in mammalian models.

This study hypothesizes that the projected Na-activated modified clinoptilolite can effectively absorb large quantities of Cd^2+^ ions in the digestive tract, possibly lowering Cd toxicity levels. Changes in both physiological and genetic parameters could signal this reduction. Clinoptilolite supplementation is expected to lead to differences in Cd bioaccumulation, hematological indices, oxidative stress, and DNA damage before and after supplementation.

## 2. Materials and Methods

### 2.1. Clinoptilolite

**Figure 2:**
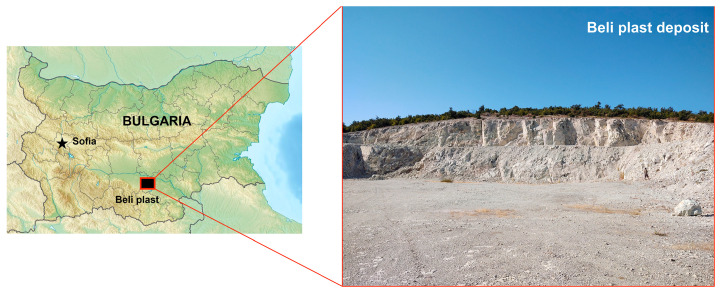
Clinoptilolite deposits in Eastern Rhodopes area near Beli Plast village, Bulgaria.

The clinoptilolite-rich tuff used in this study was collected from the volcanogenic sedimentary deposit Beli Plast, located in the Eastern Rhodopes region of Bulgaria (Figure 2). This deposit is related to the first Rupelian acid volcanic phase that occurred at the beginning of the Oligocene. In the shallow marine environment, the metastable volcanic glass transformed into more stable phases predominantly composed of clinoptilolite [12,13,14].

#### 2.1.1. Sample Collection

**Figure 3:**
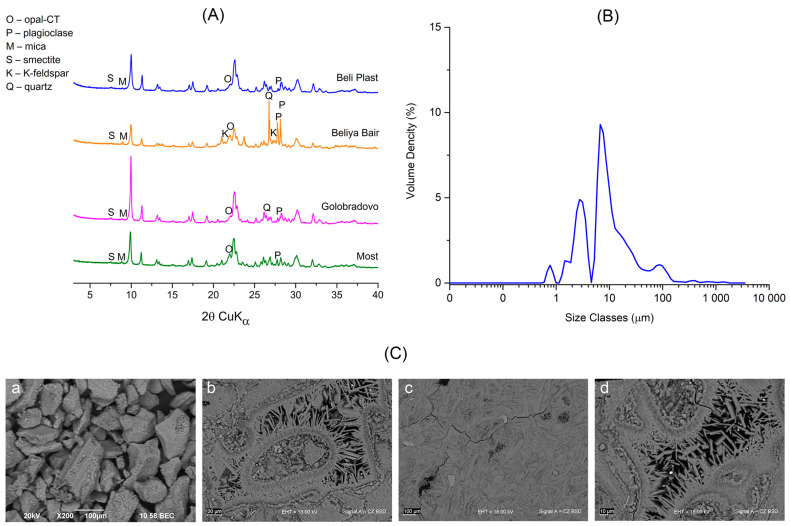
(**A**) Powder X-ray diffraction patterns of studied clinoptilolite-rich tuffs. Minerals associated with clinoptilolite depicted. (**B**) Mie sizing of tribo-activated Na-exchanged clinoptilolite. (**C**) Back-scattered electron images (BSE) of used fraction < 0.15 mm (**a**); poorly crystalline clinoptilolite (**c**) and prismatic crystals up to 30 μm long (**c**,**d**) (**b**,**d**—polished sections).

Samples of clinoptilolite-rich tuffs from the four major Bulgarian deposits—Beli Plast, Golobradovo, Most, and Beliya Bair—were collected in order to identify a suitable material for our clinoptilolite sorbent. Their mineral and chemical compositions were analyzed to select a material for use in eco-toxicological experiments as a sorbent combined with traditional food (Table S1, Figure 3A).

European legislation allows a limited concentration of toxic metals in clinoptilolite tuffs used as food additives [51]. EU regulations [52] permit using sedimentary clinoptilolite as a food additive. They authorize using zeolitized tuff if the clinoptilolite content is ≥80%, clay minerals are ≤20%, and it is free of fibers and quartz. According to these regulations, the clinoptilolite-rich tuffs from the Beli Plast deposit were found to be particularly suitable for use as a mineral additive since their levels of Pb and Cd were below the maximum permissible values of 60 ppm and 5 ppm, respectively. The concentration of As was under the detection limits (Table S1). The tuffs contained >80% clinoptilolite, <20% montmorillonite, and were free of quartz (Figure 3A).

#### 2.1.2. Preparation of Na-Exchanged Clinoptilolite Sorbent

The ion exchange method is regarded as relatively cost-effective and efficient for removing hazardous metals. According to [53], Na-exchanged clinoptilolite has a greater Cd exchange capacity than the natural form. To prepare a sorbent for detoxification, clinoptilolite tuff from the Beli Plast deposit was ground and sieved, with the fraction below 0.15 mm being separated for treatment. The Na-exchanged sample was prepared by stirring 100 g of the natural sample with 1000 mL of a 1 M NaNO_3_ (Merk Cat. No 104711) solution at 500 rpm. The experiment was performed at 80 °C in a closed bottle. The NaNO_3_ solution was replaced daily with a fresh one. This procedure was repeated 12 times, after which the sample was washed with deionized water and dried at 60 °C.

After Na exchange, the sample was mechanically tribo-activated using the ball-mill method to enhance the selective properties of the clinoptilolite by reducing its particle size and increasing the surface area of the zeolite material.

#### 2.1.3. Characterization Methods

Powder X-ray diffraction (PXRD) analysis was conducted using a PANanalytical EMPYREAN diffractometer(Malvern Panalytical, Almelo, The Netherlands) with a goniometer radius of 240 mm. The system operated at 40 kV and 30 mA, utilizing CuKα radiation, and included a 3D pixel detector. PXRD measurements were taken at room temperature across the 2θ range of 3–70° with a scanning rate of 0.013° over 80 s.

The chemical composition of the major elements was determined using inductively coupled plasma optical emission spectrometry (ICP-OES). Trace element contents were obtained through laser ablation inductively coupled plasma mass spectrometry (LA-ICP-MS) with a PerkinElmer ELAN DRC-e ICP-MS (PerkinElmer, Concord, Canada) and a New Wave UP193-FX excimer (ATLEX-LR, Wermelskirchen, Germany) laser system. The element concentrations represented the average values derived from three measurements of pellets prepared with lithium tetraborate at 1050 °C.

Scanning ZEISS EVO 25LS electron microscopy (Carl Zeiss SMT Ltd., Cambridge, UK)) equipped with an EDAX Trident system, operating at a 16 kV accelerating voltage and an approximately 1 nA beam current, with reference standards and electron probe microanalysis (EPMA) were utilized to analyze the morphology and chemical composition of individual clinoptilolite grains. 

The particle size distribution of the clinoptilolite powder was performed using laser diffraction and the wet dispersion method. The tests were conducted using a Mastersizer 3000 Malvern-Panalytical device (Malvern Panalytical, Almelo, The Netherlands). The size of particles smaller than 6 μm in the Na-exchanged and tribo-activated clinoptilolite was obtained using the Dynamic Light Scattering (DLS) technique (Brookhaven Instruments Corporation, New York, NY, USA). The particle size distribution was determined at 657 nm and 90 degrees using Dynamic Laser Scattering measured by a goniometer from Brookhaven Instruments Corporation (New York, USA), Bi-90 plus, equipped with a correlator and an avalanche photodetector (APD) (Brookhaven Instruments Corporation, New York, USA). The particle size measurement ranged from 1 nm to 6 µm and conformed to [54] standard. The measurements for the tribo-activated particles of clinoptilolite in a diluted aqueous solution were conducted at 25 °C, with an average count rate of 396.03 kcps in 3 mL disposable polystyrene cuvettes. The measurements were performed in triplicate (one run consisted of three cycles of 3 min). The data were analyzed using the Particle Solutions (version 3.6.0.7136) software.

The textural parameters of the clinoptilolite powder, including the specific surface area and pore volume, were measured using nitrogen adsorption/desorption at −196 °C with the Micromeritics 3Flex surface characterization analyzer (Micromeritics, Norcross, GA, USA). Before the test, the samples were outgassed at 200 °C to attain a constant mass. The specific surface area was calculated using the Brunauer–Emmett–Teller (BET) method.

### 2.2. Experimental Design

The present study was a one-way, four-variant experiment using ICR (CD-1) mice. The four experimental variants were as follows: control group (C) mice had ad libitum access to a nutritionally complete solid conventional rodent food free of Cd; clinoptilolite group (Z) mice were administered a standard rodent food supplemented with 12.5 wt.% powdered modified natural clinoptilolite; Cd group (CDw) mice received an oral solution of 0.00125M Cd(NO_3_)_2_ (Merk Cat. No 25154), dissolved in deionized water and administered through their drinking water while maintaining a standard rodent diet; and the Cd + clinoptilolite group (CDwZ) mice were orally administered a 0.00125M Cd(NO_3_)_2_ solution and provided with standard rodent food containing 12.5 wt.% powdered modified natural clinoptilolite. The Cd(NO_3_)_2_ concentration was considered to be an intermediate dose that was environmentally realistic, supported by previous studies that simulated the exposure of mammals and humans in industrially polluted areas to Cd contamination through food or water [55,56]. The four experimental variants were conducted over 45 days, during which the body mass of the animals and the necessary blood samples were collected from each experimental group at the exact sampling times for each variant at days 0, 15, 30, and 45. The obtained animal body mass was then compared to the established norms for the ICR strain of laboratory mice of the same age and sex [57]. The total sample size was calculated before the experiment, estimating an effect size of 0.4, a probability level (α) of 0.05, and a power (1-β) of 0.95. Eight mice were chosen to satisfy the statistical requirements for a small group at each time point. Each group had 32 mice, leading to an overall sample size of 128. Due to severe distress and physiological imbalance, two animals from two groups were removed, decreasing the number of ICR mice to 126. On day 30, the CDwZ group included 7 animals, while the CDw group had 7 animals on day 45.

The mice were randomly assigned to the treatment groups via a random number generator, using block randomization with a block size of 8 for equal group sizes. The treatment order within each block was determined using Excel’s RAND function. Blinding was performed during the experiment concerning group allocation, along with the outcome assessment and data analysis.

### 2.3. Experimental Animals and Husbandry

Only healthy ICR (CD-1) mice bred in the certified vivarium at the Institute of Neurobiology, Bulgarian Academy of Science, with health records and no signs of illness or injury, were included in the study. Male mice aged 6 to 8 weeks, weighing 25 to 30 g, were used to ensure group homogeneity. The mice were housed in individual, well-ventilated plastic cages (Tecniplast^TM^ ULTEM^TM^, Cat. No 02-934-122, Milano, Italy) and randomly assigned to shelf positions in the Institute of Biodiversity and Ecosystem Research vivarium following European standards (Directive 2010/63/EU) [58]. Their bedding material (GOLDSPAN^®^pellets) was sourced from an ISO 2000-accredited supplier (Brandenburgs Unternehmensgruppe, Goldenstedt, Germany). Before the experiments began, the mice were acclimatized for seven days. The study was maintained within a controlled environment at a standard temperature between 20 °C and 22 °C, a 45–60% humidity range, and a 12 h light/dark cycle. The mice were given standard pelleted rodent food and had access to food and water ad libitum. Their water, food, and bedding materials were checked daily, and replacements were made as needed. The animals were neither given medication nor vaccinated. Animals showing severe distress (e.g., persistent vocalization, self-mutilation), losing over 20% body weight, illness, or abnormal behavior affecting data were excluded due to their potential impact on the physiological parameters studied. All the experiments were conducted following Ordinance No. 20/01.11.2013 on the protection and welfare of animals and the Animal Protection Act of 31 January 2008 of the Republic of Bulgaria and approved by the Institute of Biodiversity and Ecosystem Research Ethical Committee.

### 2.4. Heavy Metal Loading

Cd concentration was determined using a Perkin Elmer SCIEX DRC-e ICP-MS system equipped with a cross-flow nebulizer. The spectrometer (RF, gas flow, lens voltage) was optimized to yield minimal CeO^+^/Ce^+^ and Ba^2+^/Ba^+^ ratios while maximizing the intensity of the analytes. Working standard solutions of Cd, within a concentration range of 0.01 to 500 µg L^–1^, were prepared from a single-element calibration solution (Merck KGaA, Darmstadt, Germany) with an initial concentration of 1000 mg L^–1^. External calibration was conducted for isotopes ^111, 112, 113, and 114^ Cd. The calibration coefficients for all the calibration curves were at least 0.99. The evaluation was performed by analyzing two certified reference materials: IAEA-H-8 horse kidney and NIES N6 CRM mussel. The calibration and sample analysis results were consistent with the certified values.

### 2.5. Weight and Organ Index Assays

Throughout the treatment period, the body weight of each mouse was measured at every experimental time point, and any changes were documented. The liver and kidneys were weighed accurately to determine the organ indices, which were calculated as follows: organ index (%) = average weight of the organ/(average body weight) × 100%.

### 2.6. Hematology

Blood samples were collected from the tail vein into 0.5 mL MicroTube EDTA K3 microtubes (Merk Cat. No 65501-24-8) and were automatically analyzed using a MindRay BC-30Vet automated veterinary hematology analyzer. The following parameters were chosen as the most representative for research purposes: red blood cell (RBC) and white blood cell (WBC) counts, hemoglobin (Hb), hematocrit (Hct), the mean corpuscular volume (MCV) of the RBCs, and the mean corpuscular hemoglobin (MCH) in the RBCs.

### 2.7. Oxidative Stress Markers

Two markers for oxidative stress were selected for analysis: (1) the presence of malondialdehyde (MDA) in organ homogenates, measured by the thiobarbituric acid-reactive substances (TBARS) test, serving as a biomarker for lipid peroxidation, and (2) the total amount of glutathione (GSH) in the same organ homogenates. The TBARS test was conducted as described in [59]. Mouse liver and kidneys were homogenized and subjected to the procedure described. MDA levels were measured spectrophotometrically by absorbance at 532 and 600 nm and expressed as nmol MDA/g of fresh weight. Total glutathione was assessed calorimetrically, following the protocol outlined in [60]. Glutathione levels were presented as mmol GSH/g of tissue.

### 2.8. Micronucleus Test

To evaluate the potential for genotoxic effects caused by exposure to environmentally relevant Cd(NO_3_)_2_ concentrations, an in vivo micronucleus test was conducted using a slightly modified version of the original acridine orange (Merk Cat. No 113000) staining technique [61]. Instead of phosphate-buffered saline (PBS), Sörensen’s sodium phosphate buffer (pH 7.4) was used. Image acquisition and analysis took place on an OPTIKA B-383 FL fluorescence microscope at a magnification of 400×, employing a blue filter set (excitation wavelength 460–490 nm, emission at 515 nm). Micronuclei (MNs) were categorized as such when they showed a similar yellowish-green staining pattern and focusing configuration as the main nucleus, exhibited a round or oval shape, and did not exceed one-third of the main nucleus’s size. The mean frequency of the recorded micronuclei refers to the number of cells with MNs per 2000 counted erythrocytes, expressed per thousand (‰).

### 2.9. Statistical Analysis

A power F test analysis for one-way ANOVA fixed effects was performed before the experiment commenced using the G*Power software program, version 3.1.9.4 (Heinrich-Heine-University Düsseldorf, Germany) to compute the estimated effect and sample size. The statistical significance of the mean differences between the tested parameters was calculated using Prism software, version 9.0 (GraphPad Software, San Diego, CA, USA). Both univariate and multivariate statistical analyses were conducted. The variability of the parameters under examination was evaluated through univariate statistical analysis. The results were presented as means ± standard deviation. To ascertain the normality of the data and the homogeneity of variance, the D’Agostino–Pearson and Levene F-tests were employed. The normality of the hematological data was assessed (*p* < 0.001), and a one-way ANOVA was conducted, followed by Tukey’s multiple comparison post-test. A nonparametric analysis was necessary because the data on MNs did not meet the normality test requirements. As a result, the Kruskal–Wallis nonparametric multiple comparison test with Dunn’s post hoc test was utilized. The significance level was set at *p* ≤ 0.05.

## 3. Results

### 3.1. Characterization of Natural and Na-Exchanged Clinoptilolite Tuff

The mineral composition of the clinoptilolite-rich tuff from the Beli Plast deposit was determined using PXRD. The mineral quantities obtained from the sample studied are as follows (in wt.%): clinoptilolite, ~86%; opal-CT, ~7%; mica(10Å), ~2%; montmorillonite, ~2%; and plagioclase, ~2% (Figure 3A).

The chemical composition of the major elements (in wt.%) for both the natural and Na-exchanged samples is presented in Table 1. The Na_2_O content significantly increases after sodium exchange, reaching 4.26 wt.%, while the values for the oxides of the exchangeable cations (CaO, K_2_O, and MgO) decrease.

The contents of 63 trace elements were measured to screen for toxic and potentially toxic elements in the clinoptilolite tuff. The element concentrations were converted into true values through internal standardization (concentration of Si, determined by ICP-OES). The concentrations of the trace elements were compared to the average concentrations for the upper continental crust [62]. The contents of the elements As, Cd, Co, Ge, Se, Mo, Te, Eu, Pd, Re, Rh, and Au are below the detection limits (Table S1). The LA-ICP-MS data reveal that the clinoptilolite tuff is relatively depleted in Sc, V, Cr, Ni, Cu, Zn, Ga, Y, Zr, Ba, Hf, W, and REE (except for lanthanum, thulium, and ytterbium). Relative enrichment is found for Cs, Ta, Tl, Pb, Bi, Th, U, Be, S, Rb, Sr, Nb, In, Sn, Sb, and Ag [63]. Regarding the contents of the toxic elements, the clinoptilolite-rich tuff from the Beli Plast deposit is suitable for usage as a sorbent for detoxification purposes because its contents of Pb and Cd are lower than the maximum permissible values, and its concentration of As is below the limit of detection (Table S1).

The morphological characteristics of the clinoptilolite-rich tuff from the Beli Plast deposit are shown in Figure 3C. Typically, clinoptilolite forms fine crystals (<5 μm) and pseudomorphically replaces both pumice and shards (Figure 3C(a)). It rarely occurs as prismatic crystals up to 30 μm long in the central hollows of the glass shards (Figure 3C(b–d)).

The electron probe microanalyses show slight variations in the chemical composition of the studied clinoptilolite grains (Table 2). The crystal-chemical formulas are calculated based on 72 oxygens. The Si/Al ratios determined by EPMA range from 4.22 to 4.71.

The effect of milling on the Na-exchanged clinoptilolite particles, as observed by the laser diffraction particle size analyzer, is shown in Figure 3B. The results indicate that the tuff has a grain size ranging from approximately 0.6 μm to 200 μm. Clinoptilolite particles are multimodally distributed, with the following main peaks: from 0.5 to 1 μm, from 1.5 to 4.5 μm, from 5 to 45 μm, and from 65 to 150 μm. The grains with sizes between 5 and 45 μm are the most dominant.

The distribution of particles smaller than 5 μm in the Na-exchanged and tribo-activated clinoptilolite was obtained using the DLS technique. The correlation function provides a mathematical description of the fluctuations of the scattered light. This function is utilized to assess the quality of the analyses (Figure S1). The primary parameters influencing data quality are the average hydrodynamic diameter of the particles (effective diameter) and the width of their distribution, known as polydispersity (Table S2). Figure S1 presents the scattering intensity correlation function of tribo-activated clinoptilolite water solutions at three equal concentrations and a scattering angle of 90°. The continuous lines represent the best non-linear least-squares fits obtained under the assumption of a multimodal decay of correlations.

After determining the mean particle diameter and polydispersity values, these were fitted to a lognormal distribution representing the size distribution. They interpolated cumulative and differential results at 5% intervals (Figure S2a). The cumulative distribution function of a random variable describes the distribution of that variable. Multimodal particle size distributions were established using a numerical algorithm and Mie theory. The particles were measured in the 100 to 5000 nm range, exhibiting a bimodal size distribution (Figure S2b). The finer particles display a broad size distribution in the 240–290 nm range, with a peak (average) near 260 nm. Large particles of tribo-activated clinoptilolite are observed, with a distribution that has a high-intensity peak around 1600 nm. According to the results, the larger particles have an average effective diameter of 1696.6 nm and a narrow size distribution range from 1600 to 1800 nm (Table S2).

The specific surface area of the Na-exchanged and tribo-activated clinoptilolite was calculated using the Brunauer–Emmett–Teller (BET) method. The results are presented in Figure S3. The total pore volume is 0.051 cm^2^/g, and the surface area is 24.2 m^2^/g.

### 3.2. Cadmium Bioaccumulation

The concentrations of Cd in the livers and kidneys of mice from the C and Z groups were below the detection limits of the method used. However, the Cd^2+^ accumulation levels in the organs and feces of the CDw and CDwZ mice were significantly different from the baseline measurements (*p* < 0.0001) (Table 3). By the end of the study, the CDwZ group exhibited a notable decrease in liver Cd accumulation (*p* < 0.001), ranging from 19% on day 15 to 48% on day 45 compared to the CDw group. This reduction spanned from 26% to 61% in the kidneys during the same timeframe (Table 3). Additionally, the quantity of Cd^2+^ absorbed by the modified clinoptilolite and then excreted in feces rose from 28% to 36% in the CDwZ group. This increase suggests that clinoptilolite plays a significant role in aiding the excretion of around 36% of ingested Cd through fecal output.

### 3.3. Growth Rate

**Figure 4:**
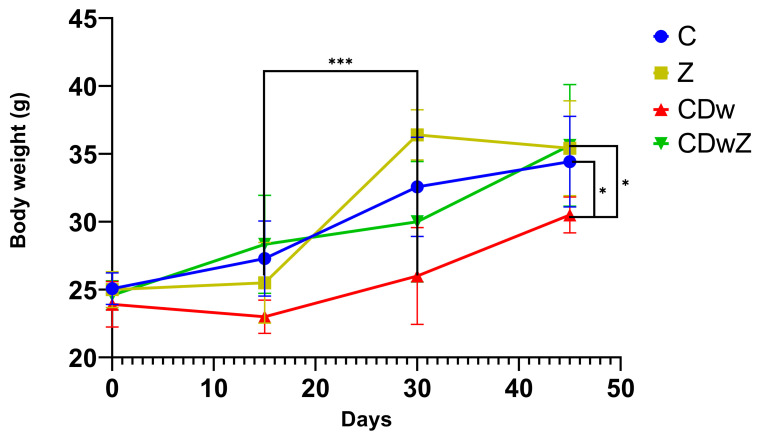
Changes in body weight (g) of laboratory mice (mean ± SD) related to experiments. Asterisks indicate statistically significant differences at * *p* ≤ 0.05 and *** *p* ≤ 0.001.

Growth trends in mouse body mass were monitored during each trial (Figure 4). By the end of the trials, all the experimental mice had gained weight. The groups supplemented with clinoptilolite showed the greatest percentage increases, with gains of 41.68% for the Z group and 42.52% for the CDwZ group. The C group achieved a weight gain of 37.34%, while the CDw group recorded the lowest, at 22.0%. Mice in the C, Z, and CDw groups had experienced some weight loss at day 15, followed by a notable rebound by day 30 that was statistically significant (*p* < 0.001). Cd toxicity severely affected the growth of the CDw mice, resulting in a significant mass reduction of 12.89% by day 45 (*p* < 0.001) when compared to the C and Z groups. The CDwZ group showed a statistically significant higher body mass at the 45th time point compared to the CDw group (*p* < 0.05). Notably, on day 45, the average weight difference between the CDw and CDwZ groups was around 5.0 g, indicating a 15% variation.

### 3.4. Organ Index Changes

**Figure 5:**
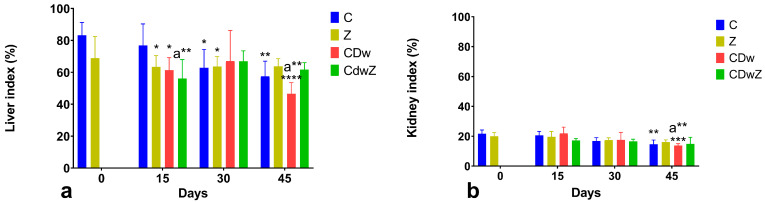
The liver index (**a**) and kidney index (**b**) for the different experimental groups. The data are presented as means ± SD. The values marked with asterisks indicate significant differences from the control group on day 0 (* *p* < 0.05, ** *p* < 0.01, *** *p* < 0.001, and **** *p* < 0.0001). The superscript (a) denotes significant differences between the control group and the other groups at the same time point.

Throughout the experiment, the liver and kidney indices exhibited similar trends. On day 45, the CDw group displayed a significant reduction in liver index (*p* < 0.0001) compared to the control group’s baseline value (Figure 5a). Additionally, by days 30 and 45, the Z and CDwZ groups experienced the most substantial healing, as their liver indices normalized, aligning with that of the control group without any statistically significant differences. As shown in Figure 5b, the kidney index for the CDw group on day 45 was significantly lower (*p* < 0.001) than that of the C group on both day 0 and day 45. Conversely, the Z group’s kidney indices did not differ significantly from the control group. The detrimental effects of Cd and the beneficial impacts of clinoptilolite were clearly evident after 45 days.

### 3.5. Hematology

The results of the hematological analyses conducted during the sub-chronic experiments, both with and without clinoptilolite supplementation, are presented in Table 4. The study showed that the WBC and MO count leucogram markers exhibited significantly elevated levels (*p* < 0.05) in the Cd-intoxicated mice compared to those not exposed to Cd from the control and clinoptilolite-supplemented groups. Administering clinoptilolite to Cd-intoxicated mice resulted in a significant decrease (*p* < 0.05) in the observed WBCs, particularly in MO, compared to the mice treated with Cd alone (Table 4). Among the erythrogram markers, the Hgb, RBC, and Hct values showed a negative effect due to Cd toxicity after the trial, indicating a significant decrease compared to the day 0 control (*p* ≤ 0.05). The erythrogram indicators suggest the presence of hypochromic microcytic anemia resulting from Cd toxicity. Clinoptilolite supplementation mitigated the adverse effects of Cd, with the mice in the CDwZ group returning to normal erythrogram levels by day 15 and maintaining those levels until the end.

### 3.6. Oxidative Stress

**Figure 6:**
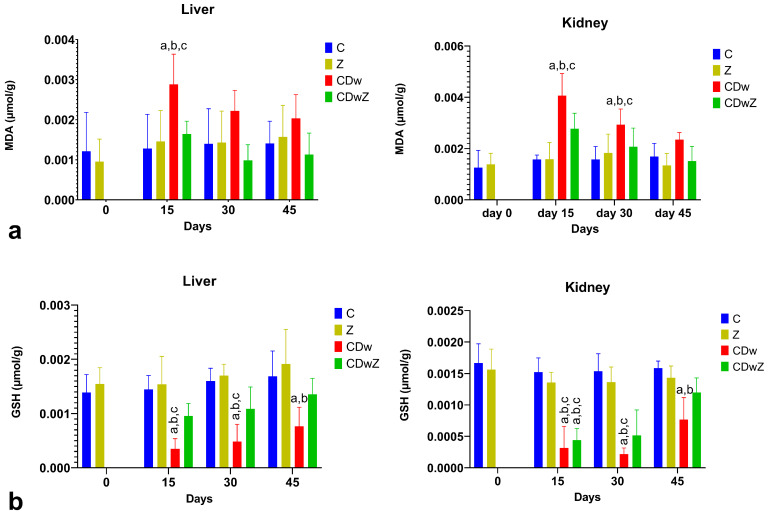
The levels of MDA (**a**) and GSH (**b**) in the livers and kidneys of the mice during various trials. The superscript (a) markings indicate statistically significant differences in the CDw mice values compared to all the control time points. The superscript (b) markings denote significant differences between the CDw mice values and all the time points in the Z group, while the superscript (c) markings highlight differences between the CDw mice and all the other time points for the CDwZ group (*p* < 0.05).

The activities of oxidant–antioxidant enzymes in liver and kidney tissues were notably affected by Cd exposure, time factors, and their combined interaction. Figure 6 demonstrates the sub-chronic hepatic and renal toxicity of Cd, assessed through GSH and MDA level measurements. Compared to the control group, MDA levels significantly increased in the CDw group (*p* < 0.05). Liver and kidney MDA contents rose significantly in response to Cd(NO₃)₂ treatment, increasing by 41.38% and 40.79%, respectively, on day 15 compared to the controls (*p* < 0.01) (Figure 6a). In contrast, antioxidant enzyme GSH levels experienced a significant decrease (*p* < 0.05) (Figure 6b). A strong inverse relationship was observed between MDA and GSH levels (*p* < 0.001, r = −0.947). On day 15, the GSH levels in the liver and kidneys were significantly diminished after Cd(NO₃)₂ treatment, falling by 185.71% and 235.04%, respectively. Thus, Cd toxicity caused an imbalance in oxidation processes in the liver and kidneys, resulting in oxidative stress. The combination of Cd and clinoptilolite treatment significantly increased GSH levels in the liver and kidneys compared to the CDw group (*p* < 0.05). However, these levels did not return to those of the control. MDA levels decreased but remained elevated compared to the control levels. By day 45, both the MDA and GSH levels were still below those of controls, with no statistically significant differences noted.

### 3.7. Micronucleus Test

**Figure 7:**
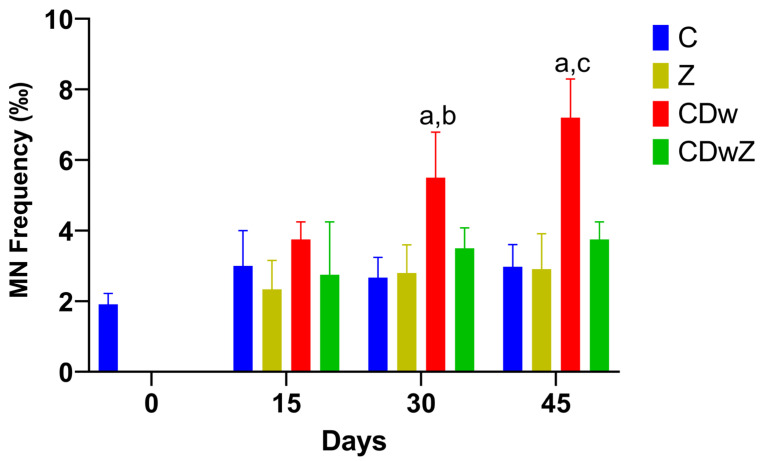
The frequency of micronuclei (MN/1000) in the erythrocytes of the mice from the C, CDw, Z, and CDwZ groups. The superscript (a) markings indicate statistically significant differences between the C and CDw groups; the superscript (b) highlights significant differences between the Z and CDw groups, while the superscript (c) denotes significant differences between the indicated CDw mice and the CDwZ mice values (*p* < 0.05).

An in vivo MN test was utilized to evaluate genotoxic stress in the red blood cells of the test animals (Figure 7). The formation of MNs in mouse erythrocytes exhibited a significant increase (*p* < 0.0001) over time following exposure in the CDw group. Conversely, no elevated MN levels were observed after clinoptilolite supplementation in the CDwZ experiment, with frequencies remaining stable and close to control levels.

## 4. Discussion

### 4.1. Sorption Properties of Modified Natural Clinoptilolite

Natural zeolites are distinctive, unique minerals often overlooked in favor of silicates in most studies. One significant type, clinoptilolite, boasts a high sorption capacity and is found in large deposits, particularly in Bulgaria. Its structural characteristics influence both ion exchange and selective sorption processes. [2]. Incorporating specific surfactants to modify zeolite surfaces has enhanced their ion exchange capacity, which aids in removing cations, anions, and various organic compounds [64]. This enhancement is essential for improving the sorption capacity of these minerals. As noted in [65], Na-exchanged clinoptilolite exhibits a higher Cd exchange capacity than its natural form.

Clinoptilolite is recognized as the only safe zeolite for medical use, owing to its well-established benefits for animal and human health and performance [65]. The European Food Safety Authority (EFSA) has issued a positive assessment of the safety and integrity of natural clinoptilolite for consumption by both animals and humans [66]. Furthermore, the EFSA showed that clinoptilolite has no toxicity in animal feed, even at high doses (10,000 mg/kg), due to its remarkable chemical properties and thermal stability. Under EU Regulation 744/2012 [51], clinoptilolite tuff from Beli Plast is suitable as a feed additive because its Pb and Cd levels are below the limits of 60 ppm and 5 ppm, respectively, with its As levels being under the limit of detection. Therefore, it may serve as a sorbent for detoxification. Previous assessments confirm that modified clinoptilolite does not cause pharmacological intoxication or unusual behavior in animals [67]. The results show that all animals given a clinoptilolite supplement survived the experiment. The mice showed increased body mass, good activity and vitality. No differences in physiological parameters were observed between the mice that received clinoptilolite and those in the control group. These research findings were solely attributed to Cd exposure and the sorption capacity of clinoptilolite under equilibrium conditions, without any influence from other stressors that might affect the results.

Limited research has documented the various in vivo effects of clinoptilolite, focused on Pb detoxification, including its antioxidant, hemostatic, immunomodulatory, and detoxification properties [18,19,20,68]. In the current study, the observed biological response in mice, indicated by the reduction in Cd accumulation in the liver, kidneys, and feces, is likely due to the porous nature of clinoptilolite and its ability to retain exchangeable cations. The theoretical internal surface area of clinoptilolite pores available for cation exchange is extensive and significant concerning the observed biological effects of clinoptilolite. Estimates range from 10 to 300 m^2^/g [69]. Similarly, the estimated surface area of the modified clinoptilolite tested in this study is 24.2 m^2^/g, slightly lower than the values (30 m^2^/g) reported for natural tribomechanically activated clinoptilolites [65].

In both humans and animals, clinoptilolite interacts within the acidic intestinal environment. The ability of fluids to reach the zeolitic surface while passing through the gastrointestinal tract relies on their specific characteristics, including the particle size of the zeolitic material, the crystallite size, the degree of aggregation, and the porosity of the individual particles [16,70]. These factors significantly impact the clinoptilolite’s ion exchange, adsorption, and catalytic properties. The SiO_2_/Al_2_O_3_ ratio in clinoptilolites is an important indicator of their acid stability. A higher SiO_2_/Al_2_O_3_ ratio correlates with increased acid stability in zeolites [17]. The Na-exchanged and tribomechanically activated clinoptilolite used in this study possesses the necessary characteristics (chemical content, grain size, multimodally distributed clinoptilolite particles, high SiO_2_/Al_2_O_3_ ratio), highlighting its suitability as a reliable sorbent for Cd^2+^.

Numerous studies have evaluated clinoptilolites’ abilities to remove heavy metals in in vitro models that simulate the stomach and small intestine conditions. The structure and sequestration capability of clinoptilolite remain unaffected by pH and dietary mixtures, indicating that the mineral can be effectively applied in these biological contexts. As employed in the present study, the concurrent administration of clinoptilolite and metal has been demonstrated in numerous studies to be efficacious when metals and clinoptilolite are combined [62,71,72,73,74,75,76,77].

### 4.2. Cadmium Toxicity and Bioaccumulation

The present study supports the previously observed effects of clinoptilolite administration on heavy metal concentration profiles in rodent organisms [16,17,18,64,67]. However, this research marks the first investigation into the effects of a specially modified clinoptilolite with an enhanced Cd^2+^ exchange capacity, used as a dietary supplement, on CDw animals. Cd has a long half-life [26] and accumulates in the liver and kidneys, potentially causing severe hepatotoxicity and nephrotoxicity [27,28]. Its absorption increases MT synthesis, forming Cd-MT complexes. Cd toxicity causes hepatocyte death, transporting these complexes to the kidneys [78], where they accumulate and heighten chronic toxicity [79]. Once reabsorbed, Cd degrades into more toxic free and bound forms, with free Cd damaging renal tubular epithelial cells significantly [28,30].

The observed decline in Cd bioaccumulation in the liver, kidneys, and excreted feces of animals from the CDwZ group demonstrates the effectiveness of the modified natural clinoptilolite’s chemical composition and morphological structure in enhancing Cd sorption capacity. By day 45, clinoptilolite had reduced the Cd content in liver tissues by about 50%, in kidney tissues by 60%, and in feces by 36%. The concentration ratio of Cd45/Cd15 is often called the bioaccumulation coefficient. The bioaccumulation coefficients in the exposed and unsupplemented mice were significantly higher than in the exposed and supplemented mice [17]. For the CDw group, the coefficients were 3.07 for the liver, 4.90 for the kidney, and 1.54 for the feces. For the CDwZ mice, they were 2.04, 2.55, and 1.77, respectively (Table 3). A significant reduction in this coefficient, particularly for the kidney, was established. This indicates that the modified natural clinoptilolite significantly reduces the Cd levels in the blood by acting within the gastrointestinal tract and considerably decreasing the Cd absorption by the mucosa. This further demonstrates the significant reduction in Cd bioaccumulation resulting from the ion exchange capacity of the Na-activated sorbent. In mice exposed to Pb, a modified natural clinoptilolite sorbent, KLS-10-MA, decreased Pb accumulation in the intestine by over 70% [17,19]. Previous studies have demonstrated that Bulgarian clinoptilolites from deposits in the Eastern Rhodopes [17,18,19] support organisms’ normal physiological state during chronic heavy metal intoxication by absorbing significant amounts of Pb^2+^. Results from several authors indicate that the Na-enriched form of modified clinoptilolite has the highest static ion exchangeability for Pb^2+^, Cd^2+^, NH_4_^+^, and others [75,76,77]. Therefore, the modification that has been performed here can be regarded as successful for detoxification.

However, further and more detailed laboratory research is necessary to clarify the effects of varying concentrations of Cd and clinoptilolite. This will enhance the understanding of this mineral’s role as a sorbent when used as an animal food additive.

### 4.3. Growth Rate

Body mass gain is a reliable indicator of an animal’s health status. The changes in this parameter after consuming zeolite-enriched food have been well-established in animal husbandry for many years [80,81,82,83,84]. Studies regarding the detoxification capacity of natural clinoptilolite following sub-chronic Pb intoxication have also shown significant alterations in body weight [17]. However, the degree of enhancement effects is influenced by the type of clinoptilolite used, its purity, its physicochemical properties, and the level of dietary supplementation [83]. Administering clinoptilolite as a food additive can significantly enhance the overall physiological condition of an organism. The decrease in body mass due to chronic Cd intoxication is notably more pronounced compared to cases where clinoptilolite was included in the diet. The current finding that the body mass of the experimental mice increased by about 15% in the presence of a clinoptilolite additive is consistent with previous results obtained from clinoptilolites from various deposits [83].

### 4.4. Hematology

Hematological parameters in mice indicate environmental and toxic stressors, making the hematopoietic system essential for assessing toxicity in small mammals [84,85,86]. Hb, RBCs, Hct, and WBCs are sensitive indicators of animal health, reflecting oxygen-carrying capacity and immune system status [87]. Hb and RBC erythrogram parameters are crucial for diagnosing anemia, often observed in Pb and Cd intoxication cases [88].

The study identified hypochromic microcytic anemia in CDw mice, aligning with previous research [31,89,90]. Cd intoxication resulted in decreased RBC counts, Hb, Hct, and increased total WBCs [31]. The microcytic anemia observed can lead to hemolysis due to RBC deformation [90], iron deficiency from competition in duodenal absorption [91], and renal anemia because of reduced erythropoietin (EPO) production [91]. After exposure, Cd^2+^ was attached to RBC membranes and plasma albumin, stimulating the production of MTs and ROS, which leads to oxidative stress in RBCs. [92,93]. RBC indices reflect cell characteristics and are stable measures. Cd exposure altered RBC membrane permeability and reduced intestinal iron absorption due to mucosal lesions, leading to lower hematocrit levels [94,95,96]. The increased WBC counts after Cd exposure may result from heightened free radicals, reduced antioxidant activity, immune response suppression, and systemic inflammation [97].

Supplementing the diet with clinoptilolite helped to alleviate the adverse effects of Cd on the measured hematological parameters (Table 2). The results reveal that the primary function of clinoptilolite supplements is to reduce the harmful impacts of physiological stress by lowering the overall levels of Cd [98]. Clinoptilolites, which function as ion exchangers, also contribute to specific biochemical transformations, stabilize animal homeostasis, and improve nutrient conversion, potentially raising RBC and Hb levels [20]. Clinoptilolite has a low solubility in water at physiological pH and is not absorbed into circulation from the gut [99]. Therefore, any effects on hematopoiesis are likely due to indirect mechanisms initiated in the gut. Ref. [100] studied the effects of natural clinoptilolite on mice’s hematopoiesis, serum electrolytes, and organ function. During 2 weeks of supplementation, a slight increase in Na levels was noted in mice on a clinoptilolite-rich diet. No other changes were observed in serum chemistry or peripheral blood levels of RBCs, Hb, thrombocytes, MCV, and MCH. Clinoptilolite’s ion exchange properties can affect gastrointestinal secretions’ pH and buffering capacity, improve iron absorption, bind heavy metal ions, and influence intestinal microflora [100]. As a result, the supply of clinoptilolite may have delayed or long-lasting effects on the erythron and could trigger a systemic lympho-hematopoietic response.

The scientific literature has documented the positive effects of various zeolites on blood parameters for over a decade. However, data on their ion exchange properties regarding chronic heavy metal intoxication and their impact on hematological parameters in mammals remain limited. Ref. [20] demonstrated that clinoptilolite materials directly detoxify in vivo, restoring erythrocytes in rats after organophosphate poisoning. Ref. [18] reported a 1.5-fold increase in the percentage of normal erythrocytes in the erythrograms of laboratory mice chronically exposed to Pb following zeolite supplementation. However, studies on other mammalian species have shown that clinoptilolite can effectively counteract Cd toxicity in pigs. Clinoptilolite supplementation prevented cadmium-induced iron deficiency anemia in growing swine receiving 150 ppm CdCl_2_ [101,102]. In this context, the present study concerning the Cd-detoxification capacity of clinoptilolites in a sub-chronic experiment and its impact on hematological parameters in small mammals represents a first step. The long-term effects of clinoptilolite on biological systems, including its potential toxicity and biocompatibility, must be more thoroughly investigated.

### 4.5. Oxidative Stress

Oxidative stress significantly contributes to Cd-induced organ toxicity and carcinogenicity [103]. Although Cd lacks redox potential, it raises intracellular ROS levels, causing oxidative damage to lipids, proteins, and DNA [104,105]. The liver is crucial in detoxifying drugs and chemicals [106].

The obtained data confirm the results of previous studies [107,108] on chronic Cd toxicity. This research has shown that Cd toxicity decreases the activity of antioxidant enzyme systems. This is marked by a substantial drop in GSH levels and a significant rise in MDA levels in the liver and kidneys, resulting in cellular oxidative stress. Higher concentrations of endogenous GSH were found in the liver compared to the kidney. However, the liver is highly vulnerable to oxidative damage caused by GSH depletion. When hepatic GSH levels decrease below 20% of normal, cells’ defenses against oxidative stress are impaired, potentially causing hepatic injury [109]. In rats, a decrease in hepatic GSH levels (27–34%) indicates Cd-induced hepatic injury [20]. Previous studies have shown similar GSH depletion in vitro and in vivo after Cd intoxication [20,110]. While mechanisms exist to neutralize free radicals and maintain cellular redox balance, Cd disrupts this equilibrium, leading to secondary hepatic injuries, such as inflammation, apoptosis, and liver dysfunction [111,112,113]. Although GSH levels remained low on day 45, the MDA levels in the liver and kidneys of both the CDw and CDwZ groups rose after 30 days. A strong correlation existed between MDA and GSH levels, indicating a reciprocal relationship. This suggests that lipid peroxidation, indicated by MDA, increases when the GSH levels in liver and kidney cells decrease. The current results are partially consistent with a study by [113] comparing the Pb- and Cd-induced oxidative stress profiles in the liver and kidneys of sub-chronically exposed mice. In the kidneys of the CDw mice, the highest accumulation of MDA was detected, along with a 1.5-fold GSH depletion. According to the authors, Cd toxicity causes more oxidative damage in the kidneys due to the bioaccumulation profiles of the two metals.

Suppressing oxidative stress by increasing antioxidant capacity mitigates the pathological changes induced by Cd exposure [38]. Current research suggests that sub-chronic Cd exposure modifies the liver’s antioxidant defense systems, which clinoptilolite could help to mitigate by reducing elevated MDA levels due to the depletion of GSH. Daily supplementation with modified natural clinoptilolite enhanced antioxidant capacity. Similar results were obtained by [20], who showed that micronized clinoptilolite supplementation in hepatectomized rats improved MDA levels and liver tissue antioxidant mechanisms. Cu-Zn superoxide dismutase (SOD) and GSH activity were higher in the rats supplemented with clinoptilolite. Extended supplementation also boosted the activities of glutathione peroxidase, catalase, total SOD, and total antioxidant capacity [21,22]. In mice treated with doxorubicin, micronized clinoptilolite effectively countered liver lipid peroxidation [20]. Clinoptilolite has positive immunomodulatory effects due to its interactions with microfold cells (M-cells) in the intestine [114]. M-cells can uptake nano- and submicron-particles, changing the cells’ redox homeostasis. Clinoptilolite particles can be retained by M-cells, acting locally on the tissue and not entering the bloodstream. This communication between M-cells induced by clinoptilolite enhances the immune response, stimulating IgA-producing B lymphocytes as a defensive mechanism against toxicants [115,116]. Further studies are required to fully clarify this issue, including more detailed investigations into sub-chronic Cd doses and longer-term experiments. The data suggest that sub-chronic Cd exposure modifies the liver’s antioxidant defense systems, which clinoptilolite could help to mitigate by reducing elevated MDA levels due to the depletion of GSH.

### 4.6. Genotoxic Effects

Cd causes toxicity by altering gene expression, inhibiting DNA repair, and interfering with apoptosis. It is associated with increased frequencies of micronuclei (MNs) and other indicators of DNA damage, significantly raising the number of micronucleated polychromatic erythrocytes [40,117]. MNs are evidence of mutagen-induced chromosomal aberrations, resulting from acentric fragments and lagging chromosomes in anaphase [40]. Cd may exert strong indirect genotoxic effects by interfering with DNA repair, leading to accumulated and stable DNA damage [118].

The current MN test demonstrates a parallel link between Cd levels in the body and heightened MN frequency during sub-chronic Cd treatment, indicating that cytotoxicity depends on Cd concentration in the internal organs and erythrocytes. Cd is known to be genotoxic even at low doses, including micromolar concentrations, with these effects appearing before any other observable physiological damage [119,120], resulting in chromosomal damage, as measured by the MN test. These effects increase linearly with dose [119,120]. After clinoptilolite supplementation, a statistically significant difference was observed on day 45 between the CDw and CDwZ mice. The reduced Cd impact in the zeolite-supplemented mice led to decreased heavy metal loading, as demonstrated by ICP-MS spectrometric analysis (Table 3), and the resulting prevention of MN formation.

More data are needed on the effect of zeolites on MN frequencies. Previous studies have shown that modified natural clinoptilolite KLS-10-MA from Bulgarian deposits, used as a Pb adsorber in mice, did not cause damage to their chromosomal structure and was effective [19]. Further investigations on the sensitivity of DNA stability to Cd toxicity could help us to understand genomic damage better and reveal the dependence between oxidative stress and genotoxic effects. The current results confirm the induction of MNs by low Cd doses and demonstrate the apparent genoprotective effect of the tested clinoptilolite as an in vivo sorbent of Cd^2+^ in animal intestines.

## 5. Conclusions

The clinoptilolite tuff from the Bulgarian deposit contains a mineral assemblage that renders it safe and suitable for feed additives, as it has lower levels of Pb, Cd, and As than the maximum permissible limits. The natural clinoptilolite was transformed into Na-exchanged clinoptilolite that was tribo-activated, resulting in an enhanced capacity for Cd^2+^ exchange. The environmentally relevant doses of Cd used in exposure experiments led to intoxication, resulting in significant bioaccumulation, hypochromic microcytic anemia, oxidative stress, and genotoxic damage. The 45-day in vivo supplementation with Na-modified natural clinoptilolite demonstrated a clear protective effect by reducing Cd bioaccumulation, restoring hematological indices, normalizing antioxidant levels, and preventing the induction of MNs. The protective effect of clinoptilolite may arise from its capacity to limit Cd absorption by the intestinal mucosa, prevent its entry into the bloodstream, and significantly decrease the physiological damage associated with sub-chronic Cd toxicity. These findings suggest a prerequisite and a potential long-term opportunity for such products to serve as cost-effective detoxifiers in Cd-contaminated areas.

## Figures and Tables

**Table 1:** Chemical composition of natural clinoptilolite-rich tuff and Na-exchanged sample from Beli Plast deposit.

	SiO_2_	TiO_2_	Al_2_O_3_	Fe_2_O_3_ (t)	MnO	MgO	CaO	Na_2_O	K_2_O	SO_3_	LOI	H_2_O	Total
Natural clinoptilolite tuff	71.08	0.12	10.88	0.96	0.09	1.03	2.78	0.31	2.76	0.15	9.3	2.31	101.77
Na- exchanged sample	69.42	0.14	10.63	0.82	0.08	0.58	0.9	4.26	2.46	0	9.13	3.56	101.98

**Table 2:** Chemical compositions (EPMA, wt%) and crystal-chemical formulas of separate clinoptilolite grains from Beli Plast deposit (based on 72 oxygen atoms).

Samples Oxides	P1-17	P1-19	P1-12	P1-13	P1-14	P1-15	P1-16
SiO_2_	69.07	70.74	66.90	68.19	66.97	67.88	68.46
Al_2_O_3_	12.70	12.82	13.44	13.38	13.38	12.31	12.34
MgO	0.82	0.90	0.84	0.88	0.91	0.88	0.81
CaO	3.86	3.93	4.51	4.52	4.54	3.76	3.89
Na_2_O	0.18	0.14	0.15	0.10	0.07	0.11	0.09
K_2_O	3.06	2.92	2.66	2.59	2.52	2.83	2.84
Total	89.69	91.45	88.50	89.66	88.39	87.77	88.43
	Atoms per formula unit (apfu)						
Si	29.588	29.664	29.106	29.235	29.136	29.661	29.691
Al	6.412	6.337	6.892	6.761	6.861	6.340	6.308
Mg	0.524	0.563	0.545	0.562	0.590	0.573	0.524
Ca	1.772	1.766	2.103	2.076	2.116	1.760	1.808
Na	0.150	0.114	0.127	0.083	0.059	0.093	0.076
K	1.672	1.562	1.476	1.416	1.398	1.577	1.571

**Table 3:** The cadmium levels in the liver, kidneys, and feces (mg/kg dry weight) of the tested animals from the Cd-exposed (CDw) and Cd+clinoptilolite (CDwZ) experimental groups.

Days	Organs					
	CDw ($\bar{x}$ ± SD)			CDwZ ( $\bar{x}$ ± SD)		
	Liver	Kidneys	Feces	Liver	Kidneys	Feces
0	0.3 ± 0.1	0.2 ± 0.1	0	0.3 ± 0.1	0.2 ± 0.1	0
15	56.2 ± 1.1 ^a,d^	53.1 ± 1.8 ^a^	113.0 ± 2.8 ^a^	44.2 ± 1.6 ^a^	39.1 ± 0.9 ^a^	156.0 ± 3.2 ^a^
30	96.0 ± 3.6 ^a,b,d^	104.0 ± 12.6 ^a,b^	153.0 ± 13.4 ^a,b,d^	78.0 ± 12.1 ^a,b^	74.6 ± 11.8 ^a,b^	200.0 ± 22.1 ^a,b^
45	173.0 ± 11.9 ^a,c,d^	260.0 ± 23.1 ^a,b,c^	177.0 ± 13.1 ^a,b,c,d^	90.0 ± 12.3 ^a,b,c^	100.0 ± 12.4 ^a,b,c^	276.0 ± 22.6 ^a,b,c^

The values marked with different superscript letters indicate a significant difference between the groups at each time point: ^a^ between day 0 and days 15, 30, and 45; ^b^ between day 15 and days 30 and 45; ^c^ between day 30 and day 45; ^d^ between the CDw and CDwZ groups at the same time point. The values a, b, c, and d are significant at *p* < 0.0001.

**Table 4:** The impact of Cd exposure and clinoptilolite supplementation on hematological parameters in the tested laboratory mice during the sub-chronic experiments. The data are presented as means ± SD with N = 32 for all the experimental groups.

Parameters		WBC (g/L)				LYM (10^9^/L)				GR (10^9^/L)			
	Groups	C	Z	CDw	CDwZ	C	Z	CDw	CDwZ	C	Z	CDw	CDwZ
Days													
0		4.3 ± 1.7	4.3 ± 1.7	-	-	3.1 ± 1.2	3.2 ± 1.2	-	-	2.2 ± 0.8	2.6 ± 0.9	-	-
15		3.7 ± 1.5	3.6 ± 1.7	9.6± 1.9 ^b,c^	4.6± 2.0	3.8 ± 0.8	2.1 ± 1.2	3.8 ± 1.2	2.2 ± 1.3	1.5 ± 0.4	1.1 ± 0.5	1.3 ± 0.3	0.8 ± 0.5
30		6.0 ± 1.3	7.2 ± 2.2	11.7± 2.5 ^b,c^	6.0± 1.3 ^c^	3.5 ± 1.4	3.8 ± 1.5	2.5 ± 1.8	2.8 ± 1.4	2.1 ± 0.7	2.7 ± 0.6 *	1.0 ± 0.6 ^b^	1.5 ± 0.7 *
45		6.6 ± 3.2	7.8 ± 2.1 *	8.1 ± 3.1	10.1 ± 7.4 *	4.2 ± 2.7	4.4 ± 1.4	3.3 ± 2.5	4.2 ± 1.0	4.0 ± 2.9 *	2.7 ± 0.8 *	1.9 ± 1.5	3.2 ± 1.2 *^,a^
		MO (10^9^/L)				RBC (10^12^/L)				Hb (g/L)			
0		2.6 ± 0.14	2.8 ± 0.19	-	-	3.3 ± 1.3	3.7 ± 1.0	-	-	69.6 ± 22.1	68.3 ± 19.8	-	-
15		1.1 ± 0.03	1.0 ± 0.05	12.9 ± 0.22 ^b^	2.1 ± 0.06 ^b^	3.7 ± 0.6	3.4 ± 0.5	2.3 ± 1.5	5.6 ± 1.2	54.4 ± 12.9	70.0 ± 43.5	36.3 ± 24.0 ^b,c^	45.0 ± 5.9
30		2.6 ± 0.14	0.9 ± 0.01	10.4 ± 0.62 ^b^	1.7 ± 0.03 ^b^	5.3 ± 2.0	3.9 ± 1.4	3.1 ± 1.9 *	5.7 ± 3.8	81.1 ± 29.5	79.3 ± 28.3	28.3 ± 16.6 ^b,c^	56.0 ± 25.2
45		4.1 ± 0.26	1.1 ± 0.07	12.4 ± 0.18 *^,a^	2.2 ± 0.06	0.9 ± 2.6 *	4.2 ± 1.3	1.8 ± 1.2 *^,a,b,c^	5.3 ± 3.5	82.9 ± 37.3	89.3 ± 10.6	22.7 ± 11.7 *^,b^	69.9 ± 14.4 ^b^
		Hct (L/L)				MCV (fL)				MCH (pg)			
0		0.25 ± 0.09	0.24 ± 0.09	-	-	46.8 ± 1.6	46.8 ± 1.6	-	-	15.3 ± 1.0	15.3 ± 1.0	-	-
15		0.26 ± 0.08	0.19 ± 0.06	0.22 ± 0.06	0.12 ± 0.07	48.7 ± 5.4	48.8 ± 6.5	42.2 ± 2.4 ^c^	50.0 ± 4.4	15.6 ± 1.5	15.1 ± 1.6	12.5 ± 0.3 ^b,c^	15.5 ± 1.0 ^c^
30		0.25 ± 0.12	0.19 ± 0.07	0.23 ± 0.14	0.27 ± 0.09	46.8 ± 1.6	48.2 ± 3.0	41.7 ± 4.1	44.4 ± 3.0	15.3 ± 1.0	14.0 ± 1.2	13.0 ± 1.1 ^b^	13.7 ± 0.7
45		0.26 ± 011	0.21 ± 0.05	0.24 ± 0.15	0.07 ± 0.04 ^a^	44.0 ± 4.5	50.4 ± 1.3	44.7 ± 3.5	41.9 ± 2.7 ^b^	14.0 ± 1.8	14.9 ± 0.5	13.5 ± 3.0	14.2 ± 1.3

The asterisks indicate statistically significant differences (*p* < 0.001) compared to the day 0 control within the column. Similarly, the ^a^ markings indicate statistically significant differences (*p* < 0.05) when comparing day 45 to day 30 within the same column. The data ^b^ markings represent differences within a row compared to the control value. The ^c^ markings represent differences (*p* < 0.05) within a row compared to the previous value.

## Data Availability

The original contributions presented in this study are included in the article and the Supplementary Material. Further inquiries can be directed to the corresponding authors.

## Supplementary Materials

The following supporting information can be downloaded at https://www.mdpi.com/article/10.3390/toxics13050350/s1, Figure S1: Correlation function at three measurements; Figure S2: Particle size distribution in water (a) and particle size multimodal distribution based on APD (b); Figure S3: The BET surface area and porosity analysis of the Na-exchanged and tribo-activated clinoptilolite; Table S1: Concentrations of selected trace elements in clinoptilolite tuffs from studied deposits (in ppm); Table S2: Results from statistics data at DLS measurements.
