# Supplementary material for: Oral Supplementation with Modified Natural Clinoptilolite Protects Against Cadmium Toxicity in ICR (CD-1) Mice
Source: Toxics. 2025 Apr 27;13(5):350. doi: 10.3390/toxics13050350 (PMC12116013; doi:10.3390/toxics13050350)
Supplement: Supplementary file 1 [file toxics-13-00350-s001.zip › toxics-3573661-supplementary.pdf]

Supplementary materials

Modified natural clinoptilolite alleviates cadmium-induced toxicity in ICR albino mice

Michaela Beltcheva[1*], Yana Tzvetanova[2], Peter Ostoich[1], Iliana Aleksieva[1], Tsenka Chassovnikarova[1,3*], Liliya Tsvetanova[2] and Rusi Rusew[2]

Table S1. Concentrations of selected trace elements in clinoptilolite tuffs from the studied deposits (in ppm)

Element	Deposit			
	Beli Plast	Beliya bair	Golobradovo	Most
Be	30.73	31.85	<31.001	<22.048
Sc	3.20	3.79	4.22	3.39
V	19.07	36.49	29.76	29.07
Cr	38.46	54.18	52.35	57.03
Co	<1.125	2.58	2.56	1.06
Ni	5.17	9.82	11.83	<6.518
Cu	6.74	16.31	8.77	7.10
Zn	34.76	47.14	37.44	32.79
Ge	<15.165	<13.664	<16.333	<11.996
As	<13.736	<15.416	<17.958	<12.599
Ga	13.80	15.92	17.15	15.42
Se	<13.177	<17.561	<17.433	<14.396
Y	20.25	11.98	13.28	10.69
Zr	119.94	72.49	95.91	90.43
Nb	24.62	14.72	21.64	23.09
Ag	1.68	0.96	<2.177	<1.462
Cd	<5.754	<7.940	7.23	<5.253
In	0.20	0.21	<0.384	<0.265
Sn	5.05	3.92	4.68	7.08
Sb	0.82	<1.383	<1.476	<0.943
Te	<8.892	<9.907	6.89	6.50
Cs	18.53	15.02	29.25	28.69
Hf	3.72	2.74	3.53	3.44
Ta	1.65	0.93	1.59	1.36
W	1.37	4.80	1.60	7.62
Tl	1.94	3.05	1.03	1.42
Pb	57.32	103.07	66.74	26.41
Bi	0.55	0.66	0.80	0.74
Th	32.85	29.66	22.45	22.50
U	14.21	14.12	8.76	9.57
ΣREE	134.57	119.86	113.49	106.64

Table S2. Results from the statistics data at DLS measurements

Measurements	Eff. Diam. (nm)	Half Width (nm)	Polydispersity	Baseline Index
Run 1	1797.7	1145.4	0.406	0.0 / 73.89 %
Run 2	1606.6	988.5	0.379	4.2/ 67.40 %
Run 3	1685.5	1009.1	0.358	0.0/ 71.68 %
Mean	1696.6	1047.6	0.381	1.4/ 70.99 %
Std. Error	55.4	49.2	0.014	1.4/ 1.91
Combined	1701.8	1063.3	0.390	0.0/ 70.99 %

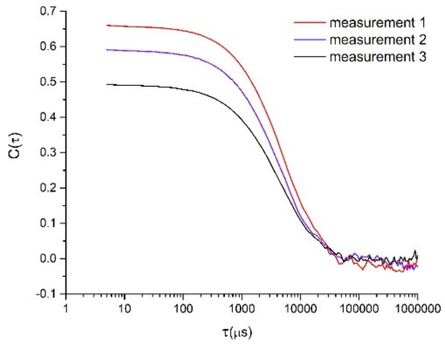

Figure S1. Correlation function at three measurements

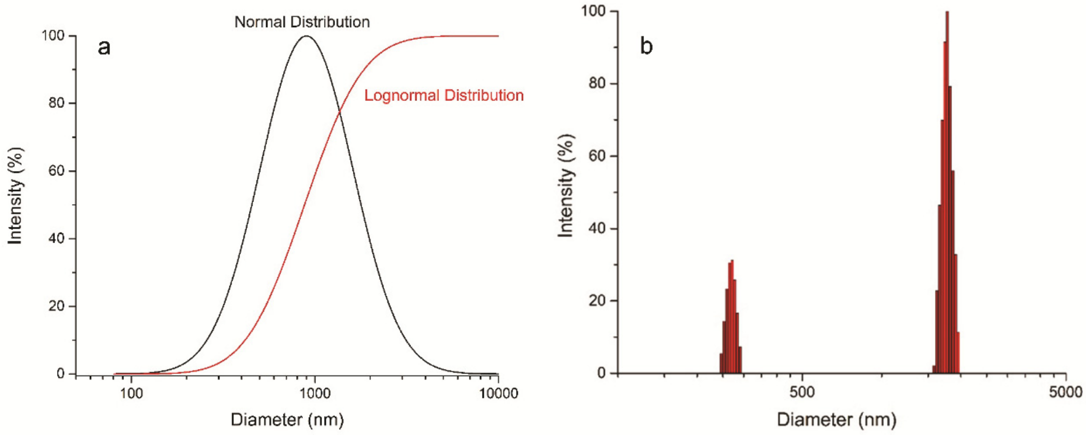

Figure S2. Particle size distribution in water (a) and particle size multimodal distribution based on APD photodetector (b)

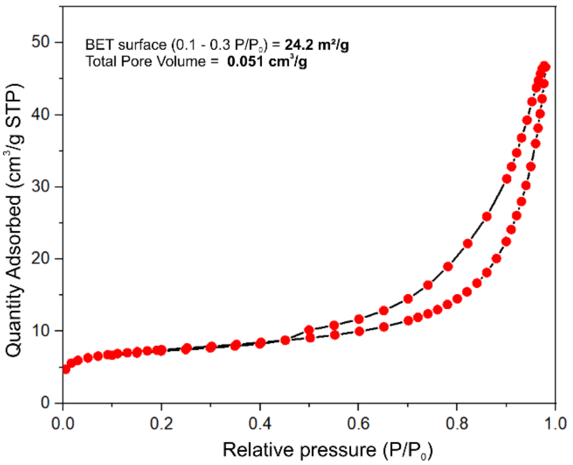

Figure S3. The BET surface area and porosity analysis of the Na-exchanged and tribo-activated clinoptilolite
